# Is Desmin Propensity to Aggregate Part of its Protective Function?

**DOI:** 10.3390/cells9020491

**Published:** 2020-02-20

**Authors:** Sonia R. Singh, Hikmet Kadioglu, Krishna Patel, Lucie Carrier, Giulio Agnetti

**Affiliations:** 1 Institute of Experimental Pharmacology and Toxicology, University Medical Center Hamburg-Eppendorf, 20246 Hamburg, Germany; s.singh@uke.de (S.R.S.); l.carrier@uke.de (L.C.); 2 DZHK (German Centre for Cardiovascular Research), partner site Hamburg/Kiel/Lübeck, 20246 Hamburg, Germany; 3 Center for Research on Cardiac Intermediate Filaments, Johns Hopkins University, Baltimore, MD 21205, USA; mzafumi1@jhmi.edu (H.K.); kpatel75@jhu.edu (K.P.); 4 DIBINEM, University of Bologna, 40126 Bologna, Italy

**Keywords:** desmin, heart failure, intermediate filaments, protein misfolding and aggregation

## Abstract

Desmin is the major protein component of the intermediate filaments (IFs) cytoskeleton in muscle cells, including cardiac. The accumulation of cleaved and misfolded desmin is a cellular hallmark of heart failure (HF). These desmin alterations are reversed by therapy, suggesting a causal role for the IFs in the development of HF. Though IFs are known to play a role in the protection from stress, a mechanistic model of how that occurs is currently lacking. On the other hand, the heart is uniquely suited to study the function of the IFs, due to its inherent, cyclic contraction. That is, HF can be used as a model to address how IFs afford protection from mechanical, and possibly redox, stress. In this review we provide a brief summary of the current views on the function of the IFs, focusing on desmin. We also propose a new model according to which the propensity of desmin to aggregate may have been selected during evolution as a way to dissipate excessive mechanical and possibly redox stress. According to this model, though desmin misfolding may afford protection from acute injury, the sustained or excessive accumulation of desmin aggregates could impair proteostasis and contribute to disease.

## 1. A Brief Introduction to Intermediate Filaments

Intermediate filaments (IFs) are an abundant and yet understudied component of the cytoskeleton in metazoans, and they are especially enriched in cardiac cells. The IFs network encompasses the cell architecture, spanning from the membrane to and within the nucleus. The IFs are divided into two subgroups based on their cellular distribution and composition: cytoplasmic and nuclear. While cytoplasmic IFs are highly heterogeneous, nuclear IFs, which are major constituents of the nuclear lamina, are mainly composed of lamins [1]. Mutations in *LMNA* encoding lamin A/C are one of the most common causes for a syndrome that strikingly recapitulates accelerated aging in humans: Hutchinson–Guilford disease or progeria (from the Greek *progeros* or prematurely old). The most common mutation responsible for progeria impacts lamin A/C maturation, leading to the accumulation of a permanently farnesylated and truncated form called progerin along with severe loss of nuclear architecture [2]. These molecular features are clinically reflected by mortality in the second decade of life, mainly due to cardiovascular disease [2]. This observation points to a key role of the IFs in one of the most compelling medical problems in our century: cardiovascular aging.

Unlike the other two components of the cytoskeleton (microtubules and microfilaments), cytoplasmic IFs are constituted of different proteins, which are expressed in a cell-type and developmentally specific fashion. For example, desmin is the major protein component of the cytoplasmic IFs in muscle cells, vimentin in mesenchymal cells such as fibroblasts, keratins in epithelial cells, etc. [3]. About 70 genes encode for IFs proteins, which can be co-expressed in the same cell-type as a function of development and disease [4]. For instance, fetal cardiac myocytes co-express vimentin and desmin [5]. While vimentin expression is rapidly reduced after birth and for most of the adult life, it can reappear as a member of a group of fetal genes that get re-expressed in dilated cardiomyopathy (DCM) and with acute rejection from cardiac transplantation [6]. In addition, Capetanaki and colleagues reported that ectopic expression of keratines 8 and 18 (K8/18) in cardiac myocytes underlies the improved phenotype of desmin knock-out (*Des^-/-^)* mice when they are crossed with mice overexpressing TNFα in a cardiac-specific fashion (*Tnf^Myh6^*). This is counterintuitive as both *Des^-/-^* and *Tnf^Myh6^* mice, taken individually, display worsened cardiac function, which is partly rescued by crossing them [7]. The presence of K8/18 in cardiac myocytes was demonstrated in this study through RNAseq data and immunofluorescence. Unfortunately, the fact that keratins are common contaminants in mass spectrometry-based studies sometimes leads to skepticism about their actual presence in a given biological sample among non-experts. The majority of IF genes encodes for keratins (~50 genes), which are fairly conserved [8]. Therefore, specific knowledge and accurate database notation are necessary to discriminate between keratins that belong to the sample from those who may belong to the experimentalist. With the advent of single cell RNA sequencing, the heterogeneity of IFs composition in the cell as a function of development and disease can now be addressed. Given the relatively poor correlation between mRNA and protein levels [9], the presence of alternate IF constituents needs to be confirmed at the protein and cell-type level (e.g., by immunofluorescence) as for the study by Capetanaki’s group on K8/18.

## 2. Desmin Loss and Gain of Function in the Heart

Desmin was first isolated from avian smooth muscle in 1976 [10]. The human protein is encoded by the *DES* gene spanning nine exons, with 8,363 base pairs resulting in a protein sequence of 470 amino acids with a size of ~54 kDa [11]. *DES* is highly conserved among species, underlining the importance of its primary sequence. Like all type III IFs, desmin has a tripartite structure with and N-terminal head, a highly conserved amphipathic central α-helical rod domain, and a C-terminal tail domains. The α-helical domain is constituted by four α-helices (1A, 1B, 2A, and 2B), which are separated by non-helical linker regions. In striated muscle cells, desmin localizes at the Z discs and its canonical role is that of maintaining myocyte ultrastructure and organelle organization [12]. However, the creation and extensive characterization of global desmin knock-out mice (*Des^-/-^*) suggest that this view may be simplistic [13,14]. Though desmin is expressed in straited and smooth muscle cells, the most prominent feature in *Des^-/-^* mice is the development cardiac myocyte hypertrophy, induction of fetal genes and prominent mitochondrial defects, which are mirrored by ventricular dilatation and compromised systolic function at the organ level [15]. While desmin is one of the most abundant protein in cardiac myocytes (~2% of the total proteome [12]) and undoubtedly the major component of the IFs in these cells, expression of other type III IF components could explain the fact that systemic desmin ablation is compatible with life in mice. In addition, since the creation of the *Des^-/-^* mice in the 1990s, a progeny that is better able to cope with the lack of desmin could have been selected over time. This view is corroborated by a recent study showing heavily impaired nuclear morphology, strikingly reminiscent of progeria’s cells, after acute knock-down of desmin via administration of viral vectors carrying a shRNA against desmin in neonatal rat cardiomyocytes and rat hearts [16]. This is not the first report highlighting the importance of the interconnection between nuclear and cytoplasmic IFs in cardiac cells. The groups of Bonne and Capetanaki recently showed that overexpression of the small heat-shock protein αB-crystallin (CryAB) partially rescues cardiomyopathy by stabilizing the desmin cytoplasmic network in H222P *LMNA* mutant mice [17]. Alpha-B-crystallin is the most abundant small heat-shock protein in the heart [18] and an established binding partner of desmin [19]. Mutations in the *CRYAB* gene can also cause desmin-related myopathies (DRMs, see below), phenocopying *DES* mutations [19]. In addition, the R120G CryAB mutant mouse, which exhibits formation of cytoplasmic desmin aggregates and dilated cardiomyopathy, is one of the most established mouse models of cardiac proteotoxicity and ideally suited to study the gain of toxic function exerted by wild-type desmin [20].

Desmin-related myopathies are a subgroup of myofibrillar myopathies caused by mutations in desmin or desmin-interacting proteins, and they often result in cardiomyopathy [21]. One of the histopathological features with the majority of *DES* mutations is the accumulation of desmin-positive aggregates in the cytoplasm of cardiac myocytes [21]. However, DRM-causing mutations are not the only cause of desmin aggregation in the heart. In fact, we first reported that the misfolding of wild-type desmin leads to the formation of desmin-positive aggregates in endothelin-1-hypertrophied neonatal rat ventricular myocytes [22]. In addition, we demonstrated the accumulation of desmin-positive preamyloid oligomers (PAOs) and amyloid fibrils similar to those observed in the brain of Alzheimer and Parkinson patients, in small and large animal models of heart failure (HF) and in human tissue specimens, in the absence of genetic mutations [23,24]. More recently, Kedia and colleagues confirmed the amyloidogenic properties of wild-type desmin in skeletal muscle through an in-depth biophysical characterization of the desmin sequence [25]. These authors further suggested that desmin misfolding is able to propagate in a prion-like fashion. This remarkable observation begs for two main unknowns to be addressed. First, the potential role played by misfolded desmin in the crosstalk between skeletal and cardiac muscle disease. PAOs and fibrils constituted of the pancreatic protein amylin (islet amyloid polypeptide or IAPP) were detected in cardiac tissue of animal models and clinical specimens of diabetic cardiomyopathy [26]. Therefore, PAOs and amyloid fibrils can “travel” in the blood stream and spread in a metastatic fashion. If desmin PAOs were to be found in the circulation, it could mean that desmin misfolding can propagate from desmin-containing cells such as striated and smooth muscle cells to other cells in the body. Further, due to the relative small mass of the heart compared to that of the skeletal muscle, it is tempting to speculate that additional desmin misfolding in the heart could by fueled by that taking place in the skeletal muscle. The possibility that skeletal muscle can aggravate protein misfolding in the heart could be important in several cardiac conditions, potentially in HF with preserved ejection fraction, which is typically diagnosed based on exercise intolerance.

**Figure 1:**
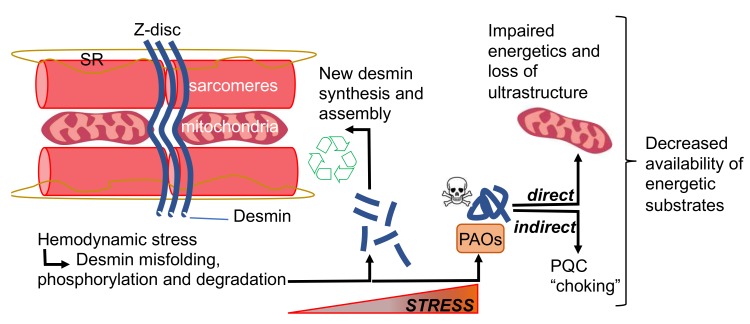
Effects of desmin misfolding on energetic substrates availability. PAOs, preamyloid-oligomers; PQC protein quality control.

From the functional standpoint, the fact that misfolded desmin can induce more desmin misfolding means that desmin gain of function and its loss of function are inextricably linked. In fact, newly synthesized desmin could be assimilated into aggregates instead of building a physiological IF scaffold in the presence of misfolded desmin. In short, the interconnection between desmin loss of function and its gain of toxic function (aggregation) makes it challenging to understand which of the two processes is more detrimental to muscle health. It is likely that both aspects contribute to cardiac dysfunction and that genetic mutations and post-translational modifications (PTMs) can bias whether gain or loss of function will represent the dominant toxic effect based on their location within desmin sequence and their extent. Ultimately, since impaired proteostasis has emerged as one of the established hallmarks of cardiac dysfunction and HF, it is likely that the misfolding of an abundant protein such as desmin would exacerbate proteostatic insufficiency [27]. In addition, beside contraction, proteostasis (protein synthesis, maintenance, and disposal) is possibly the most energetically demanding activity of the heart. Therefore, the accumulation of protein aggregates could have a taxing effect on cellular energetics, thus contributing to the increase in oxygen consumption and decrease in contractile function observed with HF. Further, PAOs and amyloid fibrils are actually toxic for cells and membranous organelles. Though the exact mechanism of cell toxicity of protein aggregates in the heart is under debate, the groups of Del Monte and Despa independently demonstrated how incubation of isolated adult cardiac myocytes with re-constituted PAOs results in the dysregulation of Ca^2+^ transients [26,28]. Specifically, while Del Monte reported that re-constituted Amyloid β PAOs induce a sudden increase in peak systolic Ca^2+^ transients and increased velocity of Ca^2+^ release [28], the group of Despa demonstrated an increase in trans-sarcolemmal Ca^2+^ leak upon treatment with amylin oligomers [26]. Suffice it to state that the dysregulation of Ca^2+^ transients would be particularly deleterious for the heart. Therefore, though tailored studies are needed to understand how PAOs exactly modify the intricate relationship between different Ca^2+^ compartments in the cell, these preliminary data confirm the importance of a general mechanism of cellular toxicity for PAOs, also in the heart. Mechanistically, the harmful effects exerted by PAOs and fibrils on ion currents, first observed in the brain, could be due to the interaction of these species, and specifically PAOs, with biological membranes and channels. It was demonstrated that PAOs can form pores in the membrane, with a specific ion conductance and susceptibility to inhibition by specific peptides [29]. Though the complete permeabilization of the sarcolemma would likely be incompatible with the survival of a cardiomyocyte, an alternative hypothesis is that PAOs could bias channels opening by directly interacting with them. Any perturbation of the tightly regulated cardiac excitation-contraction (EC) coupling could negatively affect function. In addition to the potential effects of PAOs on the sarcolemma, the sarcoplasmic reticulum and mitochondria could be also affected through their interaction with these toxic species [30]. For instance, Smolina and colleagues demonstrated that the expression of aggregation-prone desmin mutants in myotubes leads to a reduction in resting mitochondrial Ca^2+^ [31]. Interestingly, *Des*^-/-^ mice display altered mitochondrial morphology and position, which are mirrored by a decrease in maximal respiration rate and oxygen consumption which are not caused by damage to the outer mitochondrial membrane [32]. Therefore, desmin aggregation could contribute to energy starvation in HF in two ways: indirectly, by re-routing energetic substrates to the protein quality control (PQC) system; and directly, through its mitochondrial toxicity, which would result in an additional decrease in the availability of energetic substrates. Lastly, the ultrastructural defects induced by desmin loss of function are likely to affect the efficiency by which energetic substrates are delivered from the mitochondria to the sarcomere. In summary, desmin loss of function and its aggregation, which we observe in several forms of acquired HF, could play a synergistic role in contributing to energy starvation with HF (Figure 1).

## 3. Desmin and Cardiac Disease

Both skeletal and cardiac muscles highly depend on ultrastructural organization to exert their function. Therefore, desmin plays a key role in the maintenance of muscle contraction, especially in the cardiac muscle where excitation and contraction need to be tightly coupled on a beat-to-beat basis. In a seminal study, Heling et al. addressed the marked cytoskeleton remodeling which characterizes cardiac disease and reported higher levels of cytoskeletal proteins, including desmin, in end-stage HF [33]. This is one of the first studies to link cytoskeletal remodeling to contractile dysfunction in human failing hearts. The authors hypothesized that the accumulation of cytoskeletal proteins like desmin at an early stage of cardiac disease is a compensatory mechanism, which is independent from the underlying pathological trigger. When the heart decompensates, the levels of several contractile proteins decrease while the cell ultrastructure become disorganized. Since this study, many groups, including our groups, reported higher desmin levels in different forms of cardiac disease [22,23,24,34,35]. A higher levels of intact desmin could be either due to an increase in its expression or a decreased ability of muscle cells to dismantle and efficiently degrade desmin.

Regardless of the origin of the increase in desmin levels with HF, this is often accompanied by with the formation of intracellular protein aggregates. Early work from Osborn and Goebel showed the accumulation of desmin-positive inclusion bodies in a case of congenital myopathy by immuno-electron microscopy [36]. As mentioned, our group reported the accumulation of desmin aggregates in one of the most established models of in vitro cardiac hypertrophy, as well as increased desmin PAOs and short fibrils in small and large animal models of HF and both in ischemic and non-ischemic human HF [22,23,24]. Notably, ectopic expression of PAO-forming polyQ sequences are sufficient to induce cardiomyopathy in mice [37], establishing a causal role for the toxicity of cardiac PAOs. Our findings that desmin can form PAOs in the heart were recently confirmed and expanded in the skeletal muscle [25]. Specifically, Kedia and colleagues not only confirmed our previously published data on the de-fibrillization of desmin Thioflavin T-positive fibrils in the presence of the green tea polyphenol epigallocatechin gallate, but also suggested that desmin misfolding can propagate additional desmin misfolding in a prion-like fashion.

### 3.1. Desmin-Related (Cardio-)Myopathies

Desmin-related myopathies constitute a subgroup of myofibrillar myopathies that was first described as skeletal and cardiac myopathies with abnormal accumulation of desmin within muscle cells by Goebbel and colleagues in 1994 [38]. DRMs are clinically characterized by a variable penetrance and symptoms whose gravity and onset also vary among patients. One of the common features of DRMs is progressive muscle weakness that affects skeletal and potentially cardiac muscle [39]. Generally, DRMs have a poor prognosis and can be life-threatening in patients developing respiratory muscle weakness and/or cardiomyopathy. The latter is often characterized by arrhythmias and conduction defects and can result in HF and sudden cardiac death. To date, DRM patients are treated symptomatically with physiotherapy and assistive biomedical devices for skeletal myopathy. Irregular heartbeats can be managed through implantable defibrillators or pace-makers [39]. Right ventricular and dilated cardiomyopathies are most common amongst DRM patients, who present with a cardiac phenotype [21]. DRMs are mostly caused by autosomal-dominant, predominantly missense mutations in *DES* and in genes encoding proteins that interact with desmin, such as αB-crystallin (*CRYAB*) [40], myotilin (*MYO*T) [41], Z-band Alternatively Spliced PDZ-motif (*ZASP*) [42], filamin C (*FLNC*) [43], BCL-2 Associated Anthanogene 3 (*BAG3*) [44], Four And A Half LIM Domains 1 (*FHL1*) [45], titin (*TTN*) [46,47], and DnaJ homolog subfamily B member 6 (*DNAJB6*) [48]. In addition, a remarkable number of DRM cases are sporadic [39]. All of these known mutations and environmental factors lead to intracellular disarray, mitochondrial dysfunction, and defective EC coupling which are accompanied by intracellular protein aggregation in a majority of the cases [21]. Indeed, the presence of desmin aggregation is used to diagnose DRMs. *Des*^-/-^ mice do not display protein aggregates and only develop cardiomyopathy with age [13,15], demonstrating that the presence of wild-type or mutated desmin is necessary to induce protein aggregation and highlighting the importance of both desmin loss and gain of function in cardiac disease. In addition, several studies showed that upregulation of the PQC systems, such as the ubiquitin–proteasome system or the autophagy–lysosomal pathway curb the development of cardiomyopathy in mouse models of DRM [49,50]. Although the presence of intracellular aggregates is a hallmark of desminopathies, not every form of pathologically mutant desmin results in protein aggregation. For example, Taylor et al. performed a comprehensive study in 2007, in which they screened patients with dilated cardiomyopathy for *DES* mutations and overexpressed several of these mutations in IF-free SW13 cells, smooth muscle cells, and neonatal rat ventricular myocytes. This study demonstrated that not all of the mutant forms of desmin aggregate in vitro [51]. Specifically, mutations in the 1A and tail domains lead to the formation of filamentous IFs in the absence of desmin aggregates. In contrast, mutations in the 2B region, in which most disease-causing mutations were found, such as S298L, D312N, R350W, ΔE359-S361, and R406W, severely disrupted the desmin network and led to desmin aggregation. Bär and colleagues found seemingly normal filament formation with the mutations A213V, E245D, A360P, Q389P, N393I, and D399Y, and aggregate formation with mutations A337P, N342D, L345P, R350P, A357P, and L370P [52]. This body of work, combined with the direct, biophysical assessment of desmin viscoelastic properties, suggests that the N-terminal head domain of desmin is important for the correct assembly of mature filaments, while mutations in the C-terminal, tail domain are less likely to result in desmin aggregation [21]. Kedia and colleagues identified the amino acid regions 118–124 and 280–290 as highly amyloidogenic and demonstrated that the desmin fragment 117–348 can form amyloid aggregates under physiological conditions [25]. Another interesting aspect that could help our understanding of the pathology of desminopathies is what the aggregates consist of. The proteomic analysis of protein aggregates from patients with myofibrillar myopathies revealed the presence not only of desmin but also of αB-crystallin, filamin A/C, myotilin, PRAF3, RTN2, SQSTM, XIRP1, and XIRP2 [53]. In addition, proteasomal components [54], p62 [55], and αB-crystallin [56] were shown to co-localize with such aggregates, by direct inference. To our knowledge, there is no study which systematically addresses the composition of the protein aggregates in clinical specimens and in cellular and animal models of DRM. Cellular models of protein aggregation and DRM are needed in order to dissect the exact temporal sequence of events that leads to aggregation. In the majority of known proteinopathies (including Alzheimer’s and Parkinson’s diseases), PTMs and mutations lead to the accumulation of a protein seed which starts the nucleation of small soluble oligomers. These species then enter an exponential growth phase that lead to the formation of fibrils and large aggregates [30]. Due to its biophysical properties, desmin represents a likely seed for the formation of protein aggregates in DRMs and HF. In keeping with this view, the analysis of the final composition of protein aggregates (PAOs, fibrils, and large aggregates) could shed light on the mechanisms that either prevent or facilitate said aggregation. The reconstruction of the events that govern the formation of protein aggregates in the heart is key to design novel therapeutic approaches, which could be deployed in the cure of genetic and acquired forms of HF that are characterized by protein aggregation.

### 3.2. Desmin Post-Translational Processing and Misfolding in Acquired Heart Failure (HF)

The first report of the extensive alteration of desmin post-translational processing with disease comes from the early days of proteomics, when Dunn and colleagues performed extensive characterization of the proteome of human specimens from HF patients and controls using two-dimensional gel electrophoresis (2DE) [35]. Although shotgun methods, which imply the digestion of the proteome prior to its separation and analysis, became more popular in recent years, 2DE can still provide an important visual cue of the extent of post-translational processing and uniquely provide information about the cleavage of a given protein [57]. In their seminal study, Dunn and colleagues compared the proteome of 28 non-ischemic and 21 ischemic HF samples to that of 9 controls. This study revealed the induction of several different desmin proteoforms as the most prominent change associated with HF [35]. More recently, our group reported a similar induction of different proteoforms in endothelin-1-induced neonatal rat cardiomyocytes hypertrophy [22]. The acute modulation of desmin PTMs suggests that desmin post-translational processing represents a general mechanism of adaptation to stress in cardiac cells, which is conserved in human disease. We further demonstrated the accumulation of desmin aggregates in the cellular model, linking for the first time desmin post-translational processing with its aggregation. Subsequently, we addressed the causal relationship of desmin PTMs and aggregation with disease by monitoring these changes in a canine model of dyssynchronous HF (DHF), compared to sham controls and animals subjected to cardiac re-synchronization therapy (CRT). Intriguingly, the changes in desmin PTMs (mainly phosphorylation and cleavage) induced by dyssynchrony were partly reverted by cardiac re-synchronization therapy. At the same time, the accumulation of desmin PAOs was also reduced by CRT. This combined evidence suggests a strong link between desmin PTMs, its aggregation and disease in acquired forms of HF [23]. We further demonstrated that glycogen synthase kinase 3 beta (GSK3β) regulates differential desmin phosphorylation and cleavage in isolated cardiac cells. A role for GSK3β in regulating desmin phosphorylation and subsequent cleavage was recently and independently confirmed in the skeletal muscle by the Cohen’s group [58]. GSK3β typically phosphorylates substrates that are already phosphorylated and it is generally de-activated in HF samples, such as the DHF canine model [59]. Based on these available data, we proposed that accumulation of mono-phosphorylated and cleaved desmin in DHF model and in human HF specimens is a product of the inhibition of GSK3β. This is in apparent contrast with the role of GSK3β in the skeletal muscle, where the kinase primes desmin for cleavage during muscle wasting (atrophy) [58]. This could be due to the different types of cells used in the two studies (cardiac vs. skeletal) and to the fact that the signaling underlying atrophy is likely the opposite of that driving hypertrophy. While the correct timeline for the sequence of events and phosphosites involved in desmin cleavage under different conditions will require tailored experiments, the accumulation of desmin fragments could also arise from the reduced capacity of the cell to completely degrade desmin. This last possibility appears even more appealing in light of the overwhelming evidence supporting impaired proteostasis in HF. More recently, we also reported the accumulation of cleaved desmin and desmin PAOs in mouse model of pressure overload (transverse aortic constriction, TAC) and in clinical specimens from non-ischemic HF patients [24]. This combined evidence not only confirms the general occurrence and clinical relevance of desmin aggregation, but also suggests that cardiac disease can be used as a model to understand the function of desmin and of the IFs in general.

### 3.3. Using the Heart to Understand the Function Intermediate Filaments

As mentioned, the cytoskeleton in metazoans is comprised of microtubules (MTs), actin microfilaments (MFs), and the IFs. A textbook definition for the function of the MTs and MFs components of the cytoskeleton is available. While MTs are mainly used to transport various cargoes across the cell, actin filaments are primarily involved in cell motion, or contractility in specialized cells (e.g., muscle). The mechanisms through which these two structures exert their functions are established. On the other hand, IFs have been re known to play a role in the protection from stress though the mechanisms by which IFs exert their protective function are unclear. This view is widely accepted within and outside the field based on two main observations. First, insects, which possess an exoskeleton, do not express cytoplasmic IFs [8]. One way to interpret this finding is that cytoplasmic IFs appeared later on during evolution to make up for the absence of mechanical protection from stress in the absence of an exoskeleton in higher organisms. According to this view, IFs confer protection to cells that are mechanically connected to other cells within multicellular organisms and in the absence of an external “armor”. In addition to this evolutionary clue on IFs function, genetic mutations that lead to loss of function in IF proteins are usually associated with a loss of mechanical integrity. A classic example is represented by *epidermis bullosa simplex*, where mutations in keratins (mainly K14 and K15) lead to decreased mechanical resistance of the skin, resulting in severe blistering [60]. Similarly, *Des*^-/-^ mice are more prone to develop dilated cardiomyopathy [13,15].

**Figure 2:**
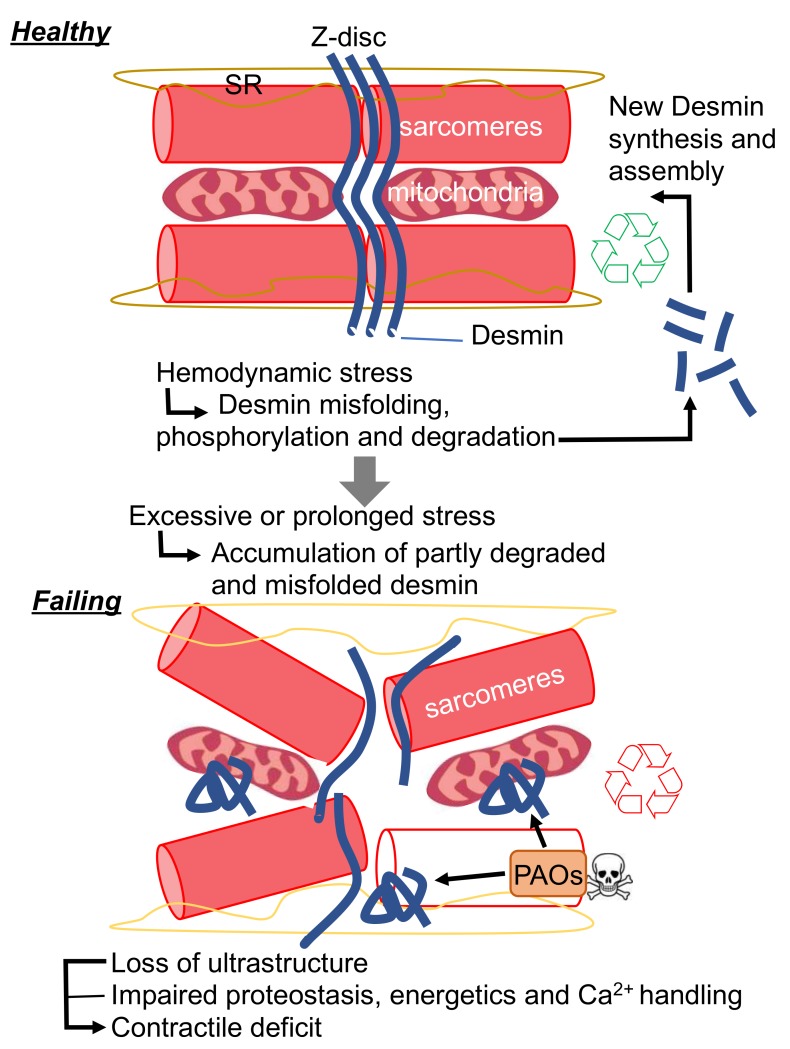
New proposed mechanism for desmin pathophysiological function in cardiac disease. SR, sarcoplasmic reticulum; PAOs, preamyloid-oligomers.

Protein aggregation is also often associated with IF-related diseases. While *DES* mutations result in protein aggregation in the majority of known DRMs, we demonstrated the general formation of desmin PAOs and fibrils in acquired forms of HF. Biophysical studies conducted on re-constituted IFs in vitro demonstrate that, unlike MTs and MFs, IFs are extremely elastic. Herrmann and colleagues were able to show that IFs can withstand up to 3–400% shear stress before breaking, while MTs and MFs are comparatively rigid. In addition, IFs become harder when subjected to strain, a biophysical property that could be at the very core of their ability to protect living cells from mechanical stress. The hagfish slime threads are largely constituted by IFs and are utilized by this ancient organism as a protection from predators. In short, the IFs were evolutionarily selected by this organism to aggregate in the presence of mechanical stress, such as that exerted by the jaws of predator [61]. Once under attack, hagfish are able to release tight bundles of IFs from holocrine glands, which are placed along side of their heel-like bodies. Once these threads are subjected to shear stress exerted by the jaws of nearby predators, their viscoelastic properties allow them to “aggregate” in a way that prevents such predators from breathing. While IF aggregation can save the hagfish life by creating a viscous matrix that chokes its predators, it is seemingly hard to reconcile why nature filled the heart with structures that react so rapidly and dangerously to mechanical stress. The peculiar viscoelastic properties of the hagfish threads are in part explained by the unfolding of α-helices to β-sheets under high strain. In fact, these properties are phenocopied by desmin IFs [62]. Therefore, it is conceivable that cardiac desmin can undergo a similar transition in the settings of cardiac disease (such as HF), where cardiac tissue, comprising individual cardiac myocytes, is subjected to pathological levels of strain. In keeping with this hypothesis, it is tempting to speculate that the propensity of desmin to aggregate could have been evolutionarily selected as a way to dissipate excessive mechanical stress through its misfolding. One of the defining features of amyloid aggregates is the enrichment in β-sheets. As such, it is possible that the desmin PAOs and fibrils that we and others reported in HF result from excessive shear stress observed with maladaptive hypertrophic remodeling. From the standpoint of homeostasis, this hypothesized mechanism could be protective from an acute increased in workload demand as observed with exercise [63]. During the phase of increased demand, desmin would “sacrifice” its folding to protect the working structures of the cardiac myocytes (e.g., the sarcomeres); while during the recovery phase, misfolded desmin could be “recycled” by the PQC system to provide regenerated desmin IFs. According to this view, the localization of desmin at the Z-discs would be ideally suited to sense and absorb excessive shear stress. Similarly, the accumulation of cleaved desmin observed with HF [22,23,24,35,64,65] could then be interpreted as a failure of the PQC system to degrade desmin completely. Partial desmin degradation would result in the accumulation of misfolded desmin that could reach the critical threshold of a seed known be able to nucleate protein aggregation (Figure 2). As we have discussed, protein aggregation would result in direct and indirect toxicity through is gain of toxic function, while concomitantly inducing loss of desmin function. The protective effects of CryAB overexpression, the potentiation of the PQC system by pharmacological intervention or exercise in R120G CryAB mice, support this model [66,67,68].

## 4. Conclusions and Future Directions

We have presented direct evidence in support of the major role played by desmin in the maladaptive remodeling that results in HF of both genetic and acquired forms. In addition, we propose that the study of cardiac desmin is not only relevant to address the increasing medical burden arising from cardiac disease, but that it also represents a unique opportunity to shed light on the elusive mechanism of protection from mechanical stress afforded by IFs. We could not address a similar role of IFs in the protection against redox stress in this review due to space constraints. However, the presence of one single cysteine which is oriented outwards in the desmin assembled tetramers, and our published data on the formation of desmin fibrils with ischemic HF [24], suggest that desmin aggregation could also protect from ischemic and ischemia–reperfusion injury. Unlike pressure overload, which represent a chronic, more subtle form of stress, heightened desmin misfolding induced by ischemia could generate aggregates that would be theoretically able to propagate desmin misfolding chronically, after the initial acute insult.

It is now critical to assess whether our hypothesis withstands the test of experimentation. New technologies comprising single cell RNAseq, novel tools to measure cardiac proteostasis and advanced mass spectrometry will allow to confirm whether increased desmin expression or insufficient proteostasis are the reason behind the accumulation of desmin and its cleaved products with HF. At the same time, new, tailored biophysical methods will enable the study of the properties of cardiac desmin to establish whether desmin misfolding can be an effective way to dissipate excessive mechanical stress. Lastly, the direct toxicity of desmin PAOs and fibrils on the membranous organelles of the cardiac myocytes, with specific reference to mitochondria, and the elucidation of the series of events that culminate in the accumulation of cardiac (and skeletal) muscle desmin aggregates, is likely to lead to the discovery of new therapeutic targets to improve outcomes in DRM and HF patients alike.
